# Challenges for Nerve Repair Using Chitosan-Siloxane Hybrid Porous Scaffolds

**DOI:** 10.1155/2014/153808

**Published:** 2014-06-17

**Authors:** Yuki Shirosaki, Satoshi Hayakawa, Akiyoshi Osaka, Maria A. Lopes, José D. Santos, Stefano Geuna, Ana C. Mauricio

**Affiliations:** ^1^Frontier Research Academy for Young Researchers, Kyushu Institute of Technology, 2-4 Hibikino, Wakamatsu-ku, Kitakyushu 808-0196, Japan; ^2^Graduate School of Natural Science and Technology, Okayama University, 3-1-1 Tsushima-naka, Kita-ku, Okayama 700-8530, Japan; ^3^CEMUC, Departamento de Engenharia Metalúrgica e Materiais, Universidade do Porto, 4200-465 Porto, Portugal; ^4^Neuroscience Institute of the Cavalieri Ottolenghi Foundation, University of Turin, Regione Gonzole 10, 10043 Orbassano, Italy; ^5^Department of Clinical and Biological Sciences, University of Turin, Regione Gonzole 10, 10043 Orbassano, Italy; ^6^Departamento de Clínicas Veterinárias, Instituto de Ciências Biomédicas de Abel Salazar (ICBAS), Universidade do Porto (UP), Rua de Jorge Viterbo Ferreira No. 228, 4050-313 Porto, Portugal; ^7^Centro de Estudos de Ciência Animal (CECA), Instituto de Ciências e Tecnologias Agrárias e Agro-Alimentares (ICETA), Rua D. Manuel II, Apartado 55142, 4051-401 Porto, Portugal

## Abstract

The treatment of peripheral nerve injuries remains one of the greatest challenges of neurosurgery, as functional recover is rarely satisfactory in these patients. Recently, biodegradable nerve guides have shown great potential for enhancing nerve regeneration. A major advantage of these nerve guides is that no foreign material remains after the device has fulfilled its task, which spares a second surgical intervention. Recently, we studied peripheral nerve regeneration using chitosan-*γ*-glycidoxypropyltrimethoxysilane (chitosan-GPTMS) porous hybrid membranes. In our studies, these porous membranes significantly improved nerve fiber regeneration and functional recovery in rat models of axonotmetic and neurotmetic sciatic nerve injuries. In particular, the number of regenerated myelinated nerve fibers and myelin thickness were significantly higher in rat treated with chitosan porous hybrid membranes, whether or not they were used in combination with mesenchymal stem cells isolated from the Wharton's jelly of the umbilical cord. In this review, we describe our findings on the use of chitosan-GPTMS hybrids for nerve regeneration.

## 1. Introduction

Nerve regeneration is a complex biological process. While approaches for peripheral nerve repair have improved over the last few decades, functional recovery is usually incomplete. As a result, much attention has been given by researchers and clinicians to cell-based therapies and tissue engineering [1–4]. Autografts are commonly used to treat peripheral nerve damage caused by accidents or diseases; however, there are several disadvantages to this approach [5, 6]. In addition, although microsurgical techniques have substantially improved, peripheral nerve repair remains one of the greatest challenges of neurosurgery [2, 4, 7, 8]. Many basic research and clinical studies have demonstrated that entubulation promotes peripheral nerve reconstruction in neurotmetic injuries with a gap, permitting reconstruction of the defect without tension at the suture and creating a favorable microenvironment at the injury site. The implanted tube guides hasten Wallerian degeneration and promote regeneration from the lesioned proximal end without the disadvantages of a graft procedure. Over the last decade, many researchers have used artificial biomaterials to produce these tube guides [9–11]. Nerve cells can regenerate into the cylinder-shaped tubes, where the microenvironment promotes regeneration towards the distal nerve stump. These tube guides can be made of different biomaterials, and they allow the incorporation of various molecules and cellular systems [12]. The tube guides should be biocompatible, nontoxic, biodegradable, permeable, and noninflammatory, like other tissue engineering scaffolds. They should prevent fibrous scar tissue invasion but allow local revascularization to improve nutrient and oxygen supply. Consequently, appropriate biomaterial microporosity is fundamental to a positive clinical outcome [13–26]. The tube guides should also have an adequate mechanical strength to maintain a stable support structure for nerve regeneration over the healing period [27].

## 2. Nerve Conduits Prepared from Chitosan-Based Materials

Chitosan and its complexes have been studied for a number of biomedical applications, including wound dressings, drug delivery systems, and space-filling implants, because of their biocompatibility, biodegradability, wound-healing, and antibacterial properties [28]. Nerve conduits derived from chitosan have favorable mechanical properties and slow biodegradability. Many chitosan nerve conduits modified with other biodegradable polymers have been investigated by numerous laboratories. Optimal hydrophilicity has been found to be essential for preventing fibrous scar tissue invasion and for promoting nerve regeneration. Cheng et al. prepared chitosan-poly(L-lysine) composites and reported that their hydrophilic surfaces improved nerve cell affinity, showing better results compared with collagen [29]. Gelatin has also been blended with chitosan to improve elasticity and enhance nerve cell affinity [30]. Cell differentiation on chitosan films is also improved by blending with gelatin. Wang et al. succeeded in obtaining regeneration across a large nerve gap of 30 mm using chitosan-polyglycolic acid (PGA) [31]. The chitosan/PGA graft-reconstructed peripheral nerve allowed the restoration of nerve continuity and functional recovery, that is, locomotion, of the operated limb [31].

Chitosan nerve tube guides have also been modified by some research groups with inorganic components. For example, Gärtner et al. prepared chitosan hollow tubes from crab tendons [32] and modified them with apatite to enhance mechanical strength, thereby preventing swelling. After implantation in a neurotmetic model of sciatic nerve injury with a 10 mm gap, the regenerated nerve tissues contained newly formed vessels, and macrophages were found to phagocytize the debris of the walls 4 weeks after surgery, demonstrating appropriate Wallerian degeneration, a crucial step toward regeneration.

Wallerian degeneration is the process of degeneration of the axon distal to the site of transection. Once a peripheral nerve has been transected, Wallerian degeneration of the distal axons begins and macrophages enter the area to remove the myelin and axonal debris. The surrounding basement membrane and Schwann cells play important roles in this process. Schwann cells line up along the basement membrane tube and synthesize growth factors that attract axonal sprouts formed at the terminal of the proximal segment of the severed axon [8]. Adding a cellular system capable of producing these important growth factors to a tube guide to direct nerve regrowth will also improve the first and essential stage of the regenerative process: Wallerian degeneration [32]. The basement membrane tubes and the artificial tube guides implanted using microsurgical techniques provide pathways for the regenerating axons to correctly innervate target muscles. The Schwann cells then remyelinate the newly formed axons; however, the newly formed myelin is thinner than normal and the newly formed internodes are shorter than normal [32].

Yamaguchi et al. [33, 34] modified chitosan/apatite composite tubes with laminin peptides to improve nerve regeneration [34]. The tubes incorporating laminin-1 and laminin peptides induced a more rapid regenerative response by improving the migration of Schwann cells and the bridging of axons. However, the recovery of nociceptive function was delayed compared with autograft treatment [34]. Chitosan-gold nanocomposite materials have also been studied and tested for use as nerve conduits [35]. The gold nanoparticles improved the mechanical strength of the chitosan. The gold in the composite affected the behavior of neural stem cells (NSCs)* in vitro*. Because NSCs are multipotent stem cells that can follow multiple differentiation pathways, using this differentiated cellular system may allow resident cell replacement and create an environment that supports and improves axon regeneration. The authors found that 50 ppm of gold nanoparticles stimulated cell proliferation and gene expression. After 6 weeks of implantation, a better and faster functional recovery was observed in animals treated with the chitosan-gold nanocomposite and NSCs compared with the composite alone in their model of neurotmetic injury with a gap. Histomorphometric analysis revealed that the number of myelinated axons in the regenerated nerve fibers was higher in animals where the nerve was reconstructed with the chitosan-gold nanocomposite and NSCs [35].

## 3. Challenges for Nerve Repair Using Chitosan-Siloxane Hybrids

Our group has synthesized inorganic-organic hybrids derived from chitosan and *γ*-glycidoxypropyltrimethoxysilane (GPTMS) units using sol-gel methods [36–42]. Different formats can be easily obtained, such as solid membranes, porous membranes, hydrogels, and tube guides. ^29^Si and ^13^C CP-MAS NMR analysis, FT-IR spectroscopy, ninhydrin assay, and contact angle analysis revealed the following physical characteristics of these hybrids [36, 38]: (1) the amino groups in chitosan reacted with the epoxy groups and interacted with silanol groups derived from GPTMS, (2) the silanol groups condensed to form 2D or 3D siloxane networks, and (3) some of the silanol groups remained in the hybrids and were orientated towards the surface of the structure.

The solid chitosan hybrid membranes allowed for good proliferation and differentiation of the human osteoblastic cell line MG63 and harvested human bone marrow cells. The attachment, proliferation, and alkaline phosphatase (ALP) activity of MG63 cells were higher when cultured on chitosan hybrid membranes compared with chitosan membranes [36, 38]. Human bone marrow cells were found to produce mineralized matrix on the hybrid chitosan membranes even without supplementation of the culture medium with dexamethasone [38], which is commonly used to improve the proliferation and differentiation of osteoblastic cells [43]. The porous hybrid chitosan membranes have interconnected pores with a size between 50 and 150 *μ*m and a very high porosity, normally more than 85% [37]. The cells adhere on the pore walls with long and numerous pseudopodia and are able to proliferate and establish connections with each other even in the middle of the porous hybrid chitosan membranes [37].

Amado et al. studied nerve regeneration using solid and porous chitosan-GPTMS membranes in a standardized crush or axonotmetic injury of the rat sciatic nerve [44]. The 3 mm long axonotmetic lesion was wrapped with solid or porous chitosan-GPTMS membranes. Functional recovery was evaluated using the sciatic functional index, the static sciatic index, the extensor postural thrust (EPT), the withdrawal reflex latency (WRL), and ankle kinematics [45–48]. Nerve fiber regeneration was also assessed morphologically using quantitative stereological analysis and electron microscopy. The porous hybrid chitosan membrane significantly improved nerve fiber regeneration in their mode, assessed using functional and morphological criteria, in comparison with untreated animals or animals treated with the solid membrane. The numbers of fibers and axons and fiber size and myelin thickness were significantly improved in the chitosan porous membrane groups (Table 1). Functional recovery also improved during the healing period of 12 weeks, as evaluated using the EPT and WRL tests. The porous hybrid chitosan membranes, which have interconnected pores, permit an adequate revascularization of the regenerating tissue and help restore metabolic communication with the surrounding microenvironment, including nutrient and O_2_ exchange [37]. These physicochemical properties help enhance nerve regeneration likely by promoting Schwann cell proliferation, neurite extension, and myelination [49–51].

Simões et al. tested the histocompatibility of the biomaterials developed for nerve regeneration by implanting the solid and porous membranes subcutaneously in the rat model. These membranes were subsequently used in experiments on sciatic nerve regeneration after axonotmetic and neurotmetic injuries [49]. The porous membranes induced a robust infiltration by multinucleated giant cells and some mast cells, whereas the solid membranes elicited mild fibrous capsule formation and a discrete inflammatory reaction. Differences in the inflammatory reaction might underlie the comparatively better regeneration obtained with the porous membranes. It should be noted that the porous membranes have a higher surface/volume ratio than the solid ones. Greater contact with the host immune system might cause the substantial cellular infiltration [49].

**Figure 1:**
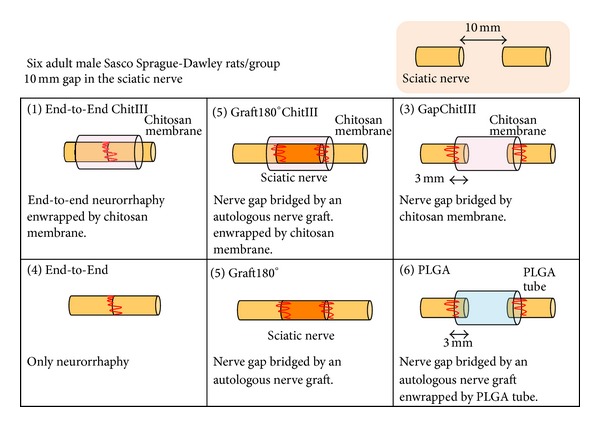
Illustration of the surgical neurotmetic repair by direct suture, autograft, or tubulization.

Simões et al. investigated the effect of the porous membranes in surgical neurotmetic repair either by direct suture, autograft, or tubulization and also compared a tube guide made of poly(lactide-co-glycolic) acid, where the two monomers, the lactic acid and the glycolic acid, were in a ratio of 90 : 10 (PLGA 90 : 10) [50]. As shown in Figure 1, the rats were divided into the following six experimental groups of six or seven animals each: Group 1, end-to-end neurorrhaphy wrapped by the porous membrane (End-to-EndChitIII); Group 2, 10 mm nerve gap bridged by an autologous nerve graft wrapped by the porous membrane (Graft180°ChitIII); Group 3, 10 mm nerve gap bridged by the porous membrane-like tube (GapChitIII); Group 4, 10 mm nerve gap bridged by an autologous nerve graft (Graft180°); Group 5, 10 mm nerve gap bridged by PLGA 90 : 10 tube guides (PLGA); and Group 6, end-to-end neurorrhaphy alone (End-to-End). After 2 weeks, the WRL test had to be interrupted at the selected cut-off time of 12 s in all experimental animals in the different groups, except the ones in the End-to-End group. The recovery of WRL was faster in the End-to-End and End-to-EndChitIII groups during the healing period of 20 weeks. After 2 weeks, EPT commenced recovery and proceeded until week 20 to a similar extent in all groups, except in the PLGA group. In the groups with the porous chitosan-GPTM hybrids (End-to-EndChitIII, Graft180°ChitIII, and GapChitIII), the EPT recovery rate was faster than in the nonhybrids groups (End-to-End, Graft180°, and PLGA). Histomorphometric analysis of the nerves was performed after the healing period of 20 weeks, which showed that a good pattern of axon regeneration occurred in all treated groups; however, the pattern of regeneration in the PLGA group was comparatively inferior. In the GapChitIII group, nerve fibers regenerated along the chitosan tube guide, which helped establish an extensive perineural connective architecture that contributed to axonal fasciculation. The neuroregenerative properties of the porous chitosan GPTMS might arise from its ability to promote the expression of myelin genes. The silica ions in the hybrid might induce expression of several glycoproteins, such as RCL, cyclin D1, and CD44. [52, 53]. Our results suggest that porous chitosan-GPTMS hybrids can be a valuable material for fashioning nerve guides aimed at bridging nerve defects.

Schwann cells, mesenchymal stem cells, embryonic stem cells, and marrow stromal cells have been extensively studied for their ability to promote nerve regeneration. We focused our research on the* in vitro* differentiated N1E-115 cell line [47–51] and on mesenchymal stem cells (MSCs) from the Wharton's jelly of the umbilical cord [32].* In vivo *experiments using the rat sciatic nerve model were performed using the porous hybrid chitosan membranes seeded with neuroglial-like cells obtained from* in vitro* differentiated N1E-115 cells. The combination of the porous hybrid membranes with these neuroglial-like cells was tested in axonotmetic and neurotmetic injuries. The implantation of cultured cells (N1E-115 cells, MSCs, Schwann cells, and other cellular systems) into defective nerves can be achieved using two different techniques. The cellular system can be directly injected into the neural scaffold that has been interposed between the proximal and distal nerve stumps or around the crush injury (in neurotmetic and axonotmetic injuries, resp.). Alternatively, implantation can be achieved by preadding the cells to the neural scaffold via injection or coculture (in most of the cellular systems, it is allowed to form a monolayer), and then the biomaterial with the cellular system is implanted into the injured nerve [47–51]. The N1E-115 cell line is a mouse neuroblastoma cell line that can undergo neuronal differentiation in response to dimethylsulfoxide, adenosine 3′;5′-cyclic monophosphate, or serum withdrawal [51, 54]. The cellular system was tested in axonotmetic and neurotmetic lesions to locally produce and deliver neurotrophic factors, which are crucial for nerve regeneration. Also, this cellular system was used as an inexpensive and easy* in vitro* cultured model of stem cells [51, 54].

Simões et al., in 2010, used the rat sciatic nerve model for investigating the effect of porous hybrid chitosan membranes in a neurotmetic injury, used together with standard microsurgical repair methods (i.e., direct epineural end-to-end suture without tension, inverted autograft, and tubulization) [50]. Morphological analysis showed that nerve regeneration had occurred when the porous hybrid chitosan membrane was used in neurotmetic injuries. At week 20, it could be observed that Wallerian degeneration was complete and followed by the regrowth of axons and Schwann cell myelinization. The results obtained with these membranes were significantly better, in terms of functional and morphological recovery, compared with PLGA 90 : 10, where regeneration was weak [50]. The favorable physicochemical properties of porous hybrid chitosan membranes compared with regular chitosan and the presence of silica might underlie their ability to enhance nerve regeneration [50]. The porous hybrid chitosan membranes might provide better mechanical support during nerve regeneration and, simultaneously, might work as an inducer of nerve regeneration, supporting the survival and ability of the Schwann cells to myelinate [50].

Light and electron microscopy analysis of PLGA 90 : 10 and chitosan tube guides containing* in vitro* differentiated N1E-115 cells suggested that the impairment in nerve fiber regeneration in these systems might have been caused by the presence of a large number of neuroblastoma-like cells colonizing large areas of the nerve profile, which might have interfered with nerve regeneration inside the nerve guides. Interestingly, the presence of many blood vessels was observed inside the large neuroblastoma-like clusters, suggesting that transplanted cells can deprive the regenerating nerve fibers of blood supply [46, 50]. It should be noted that neurotrophic factors play an important role in nerve regeneration after injury or disease, and it is conceivable that if neurotrophic factors are applied in the close vicinity of the injured nerve, their healing potency is optimized. However, contrary to our initial hypothesis, the N1E-115 cells,* in vitro* differentiated into neuroglial-like cells, did not facilitate either nerve regeneration or functional recovery in neurotmetic injuries when used in combination with PLGA 90 : 10 or porous chitosan membranes. Indeed the presence of this cellular system reduced the number and size of the regenerated fibers, thus suggesting that this type of cellular system can partially impair nerve regeneration, at least from a morphological point of view. The impaired axonal regeneration seems to be the result of N1E-115 cells surrounding and invading the regenerating nerve, since many of these cells were seen colonizing the nerve and might have deprived the regenerating nerve fibers of blood supply. Taken together, these results suggested that the N1E-115 cells do not promote nerve healing, and their use might even impair nerve regeneration. For this reason, we started using human MSCs [46, 50].

Gärtner et al. seeded the porous membranes with undifferentiated human MSCs (hMSCs) isolated from Wharton's jelly [32]. MSCs isolated from Wharton's jelly are capable of being differentiated into multiple mesodermal cell types, including skeletal muscle and neurons [55–57], and therefore may be valuable for repairing the peripheral nervous system. In the study, the axonotmetic lesion of 3 mm in the rat sciatic nerve was wrapped with (1) the porous membrane or (2) the porous membrane covered with a monolayer of undifferentiated hMSCs or (3) was directly infiltrated with a suspension of hMSCs. WRL data showed a slightly delayed recovery in the porous membrane group compared with the other groups during the healing period of 12 weeks. In contrast, EPT performance was similar in all treated groups during the healing period. Fiber regeneration was good in all treated experimental groups. The regenerated nerves contained smaller myelinated fibers than normal nerves without injury, which is expected with axon regeneration (Table 2). The myelin thickness in the regenerated nerves was higher in groups wrapped with the porous membranes alone, confirming previous results [44, 49, 52, 53], or when the lesion site was directly infiltrated with undifferentiated hMSCs [32]. The negative effects observed with the neoplastic neuroblastoma-derived N1E-115 cell line [44] were not observed with hMSCs. hMSCs enhanced functional recovery by modulating the inflammatory reaction during Wallerian degeneration and by stimulating the production of growth factors. hMSCs may have an even more pronounced effect when applied to human injures. Taken together, these findings demonstrate the therapeutic potential of hMSC and porous hybrid chitosan membranes in promoting myelin production in surgically reconstructed nerves after axonotmetic and neurotmetic injuries. These stem cells and hybrid porous chitosan membranes may also have clinical efficacy in neurodegenerative diseases that are typified by demyelination [58].

## 4. Conclusions

*In vivo* studies indicate that chitosan-GPTMS porous hybrid membranes are a very promising clinical tool in peripheral nerve reconstructive surgery. The combination of the chitosan-GPTMS porous hybrid membranes and hMSCs has a slight advantage in comparison to untreated controls. An enhancement of nerve regeneration was observed when hMSCs or the porous hybrid chitosan membranes were used alone but not when used in combination. An increase in myelin production, visible as higher myelin thickness measured by histomorphometry, was observed in surgically reconstructed nerves after axonotmetic and neurotmetic injury. These findings demonstrate the therapeutic potential of these cells and biomembranes in neurodegenerative diseases that are typified by demyelination. Further investigation using animal models is required to determine the efficacy of these systems in the treatment of critical nerve defects and neurodegenerative diseases.

## Figures and Tables

**Table 1:** Histomorphometrical assessment of the regenerated rat sciatic nerve wrapped with the solid or porous membranes during a healing period of 12 weeks after axonotmetic injury.

Group	Density (N/mm^2^)	Number (N)	Fiber diameter (mm)	Axon diameter (mm)	Myelin thickness (mm)
Control	10,123 ± 8,340	7,621 ± 198	8.27 ± 0.29	5.34 ± 0.23	1.21 ± 0.05
Crush	18,452 ± 1,952	10,180 ± 964	5.31 ± 0.34	4.12 ± 0.32	0.60 ± 0.08
Solid membrane	17,196 ± 3,364	9,774 ± 359	5.77 ± 0.45	4.54 ± 0.35	0.62 ± 0.06
Porous membrane	14,210 ± 1,600	7,780 ± 1,053	6.72 ± 0.26	5.00 ± 0.19	0.86 ± 0.05
NEUROLAC (commercial PLGA nerve guided tube) [31]	21,982 ± 1,927	10,532 ± 2,195	3.49 ± 0.11	—	0.40 ± 0.02

**Table 2:** Histomorphometrical assessment of the regenerated rat sciatic nerve, treated with hMSCs and porous membranes, during a healing period of 12 weeks after axonotmetic injury.

Group	Density (N/mm^2^)	Number (N)	Fiber diameter (mm)	Axon diameter (mm)	Myelin thickness (mm)
CrushCell	20,200 ± 4,971	9,806 ± 2,695	5.31 ± 0.19	3.74 ± 0.49	0.78 ± 0.10
CrushChCell	21,514 ± 6,308	11,413 ± 3,752	4.90 ± 0.97	3.41 ± 0.72	0.75 ± 0.14
CrushCh	15,533 ± 7,713	7,982 ± 3,092	5.29 ± 1.05	3.50 ± 0.55	1.02 ± 0.22
Control	15,905 ± 287	7,666 ± 190	6.66 ± 0.12	4.26 ± 0.07	1.19 ± 0.03
